# Prominent FLAIR Vascular Hyperintensity Is a Predictor of Unfavorable Outcomes in Non-thrombolysed Ischemic Stroke Patients With Mild Symptoms and Large Artery Occlusion

**DOI:** 10.3389/fneur.2019.00722

**Published:** 2019-07-02

**Authors:** Dae-Hyun Kim, Yoon-Kyung Lee, Jae-Kwan Cha

**Affiliations:** ^1^Busan-Ulsan Regional Cardiocerebrovascular Center, Dong-A University Hospital, Busan, South Korea; ^2^Department of Neurology, College of Medicine, Dong-A University, Busan, South Korea

**Keywords:** fluid-attenuated inversion recovery vascular hyperintensity, mild ischemic stroke, middle cerebral artery occlusion, unfavorable outcome, reperfusion therapy

## Abstract

**Background and objective:** The aim was to evaluate the clinical significance of prominent fluid-attenuated inversion recovery (FLAIR) vascular hyperintensity (FVH) on the prognosis of mild acute ischemic stroke with middle cerebral artery (MCA) occlusion.

**Methods:** We recruited consecutive stroke patients with initial National Institutes of Health Stroke Scale (NIHSS) scores ≤5 and MCA occlusion on magnetic resonance angiography within 24 h of stroke onset. Prominent distal FVH was defined as an extension to more than one-third of the MCA territory. We compared clinical outcomes between prominent and non-prominent FVH groups in patients who had and had not received reperfusion therapy.

**Results:** Of 112 participants [43 women; median age, 67 years [Interquartile range, 54–79]], prominent FVH was identified in 80 (71.4%). For 75 patients who had not received reperfusion therapy, the prominent FVH group had a more unfavorable outcome (modified Rankin Scale score >1) at 3 months than the non-prominent FVH group (44.4 vs. 15.0%, *P* = 0.029). In multivariate analysis, a higher NIHSS score [odd ratio [OR] = 1.67; 95% confidence interval [CI], 1.16–2.41; *P* = 0.006], proximal MCA occlusion [OR = 7.31; 95% CI, 1.68–31.9; *P* = 0.008], and prominent FVH [OR = 5.49; 95% CI, 1.29–23.4; *P* = 0.021], were independently associated with an unfavorable outcome. There was no association between prominent FVH and the clinical outcome in the reperfusion therapy group.

**Conclusions:** For acute stroke patients with mild symptoms and MCA occlusion who do not receive reperfusion therapy, prominent FVH and proximal MCA occlusion may be independent predictors of an unfavorable outcome.

## Introduction

More than half of all ischemic stroke patients have a clinical syndrome with mild neurological deficit (1). In spite of an initial presentation of minor deficit, a substantial proportion of stroke patients with mild symptoms become disabled (2). Large artery occlusion is an important predictor of early neurological deterioration or poor outcome in acute ischemic stroke with mild symptoms (2–4). Thus, it may be necessary to identify which patients are at high risk of an unfavorable outcome in mild stroke with large artery occlusion.

Fluid-attenuated inversion recovery (FLAIR) vascular hyperintensity (FVH) is a distal hyperintense vessel sign on FLAIR images in the subarachnoid space, presenting as absence of the flow-void phenomenon, which results from sluggish blood flow. FVH is frequently identified in acute stroke patients with large vessel occlusion (5–7), and it decreases or disappears on follow-up images after cerebral revascularization (6, 8). Thus, it may be a radiological marker of retrograde leptomeningeal collateral flow (6). However, it is still controversial whether FVH serves as a surrogate for a good collateral status or hemodynamic impairment and predicts patients' prognosis (9–17).

The clinical implications of prominent FVH in acute stroke patients with mild symptoms and large artery occlusion are not well known. This study aimed to evaluate the clinical characteristics and influence of FVH on the prognosis of ischemic stroke patients with mild symptoms and middle cerebral artery (MCA) occlusion according to reperfusion therapy.

## Materials and Methods

The data of patients who had anterior circulation stroke with mild symptoms were extracted from a prospective database of acute stroke at our tertiary stroke center between January 2012 and May 2018. In our center, the decision to treat by intravenous recombinant tissue plasminogen activator was made by stroke neurologists based on clinical guidelines. A low threshold for the National Institutes of Health Stroke Scale (NIHSS) was not applied.

We selected consecutive patients with mild acute stroke who (1) were admitted within first 24 h of symptom onset; (2) underwent emergency stroke magnetic resonance imaging (MRI) and MR angiography (MRA); (3) had positive lesions in the anterior circulation territory visualized on diffusion-weighted imaging (DWI); and (4) had MCA occlusion (proximal M1, distal M1 or M2) in MRA. We defined mild stroke as an NIHSS score ≤5. We excluded patients with (1) admission after 24 h from the last normal time; (2) unavailable or poor-quality MR images; and (3) tandem occlusion on MRA.

Demographic, clinical, and laboratory data were collected from our stroke registry. During this period, MRI was systematically implemented in our center as the first-line imaging modality (DWI, FLAIR, gradient echo sequences, and time of flight MRA) for acute ischemic stroke. In the absence of contraindications, all patients underwent pretreatment MRI using a 3.0T MRI system (GE Medical Systems, Milwaukee, WI). This study was approved by the Institutional Review Board of Dong-A University Hospital.

### Clinical Assessment

Outcome measures were early neurological deterioration (END) and the modified Rankin Scale (mRS) at 3 months from stroke onset. END was defined as an increase of 2 or more points in the NIHSS scores between hospital days 0 and 5. Clinical outcome at 3 months was dichotomized into favorable (mRS 0–1) and unfavorable (mRS 2–6).

### Imaging Analysis

**Figure 1:**
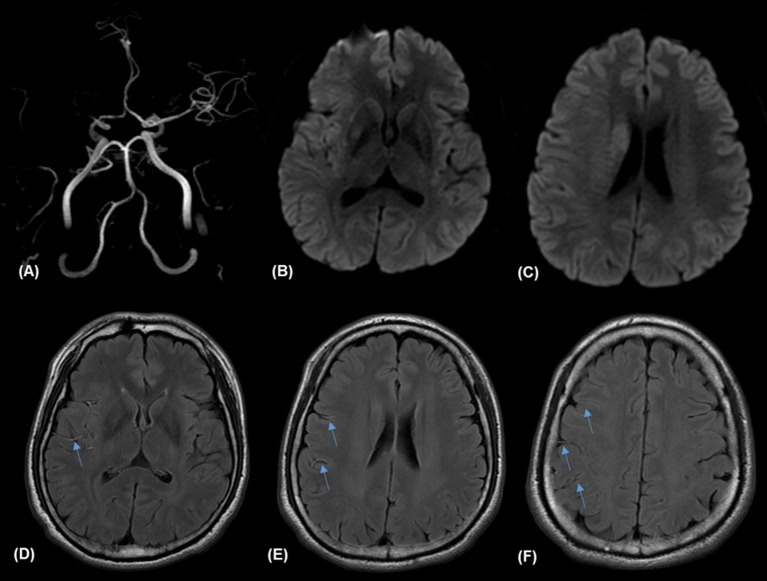
Illustrative case of prominent fluid-attenuated inversion recovery (FLAIR) vascular hyperintensities (FVH) and FVH-diffusion-weighted image (DIW) mismatch. Magnetic resonance (MR) imaging of a 57-year-old man obtained 138 min after sudden onset of left hemiparesis. Right proximal middle cerebral artery (MCA) occlusion on MR angiography **(A)** and small hyperintense lesions in the right MCA territory on admission DWI **(B,C)** with prominent FVH on FLAIR **(D–F)**, which is more extensive beyond the boundaries of the DWI high signal area, indicating an FVH-DWI mismatch.

Two investigators (D-HK and Y-KL), who were blinded to the patients' clinical characteristics, reviewed FLAIR images at admission. Discrepancies were resolved by consensus. FVH was considered as a focal, tubular, or serpentine high-signal intensity distal to the occlusion on FLAIR images into the subarachnoid space (6, 7). Prominent distal FVH was defined as an extension to more than one-third of the MCA territory (Figure 1) (7). FVH burden was divided into two groups based on the presence of prominent FVH. The presence of FVH-DWI mismatch was also investigated, and was considered present when FVH extended beyond the boundaries of the cortical DWI lesion (12). DWI lesion volume was measured using a Picture archiving communicating system (PACS) workstation (Marosis, Marotech). The area of each regions of interest was multiplied by the section thickness plus the intersection gap and then summed to give the lesion volume. The occlusion site related to lesions were determined using the initial MRA, which included the M1 proximal, M1 distal, and M2 segment of MCA. Arterial occlusion was defined as a complete loss of distal flow signal. The M1 segment of the MCA was divided into 2 parts of equal length: the proximal and distal halves.

### Statistical Analysis

All continuous variables are presented as mean ± standard deviation or median (interquartile range [IQR]) and were compared using the Student's *t*-test or Mann-Whitney *U*-test, depending on the normality of data distribution. Categorial variables were compared by using the chi-squared or Fisher's exact test as appropriate.

We analyzed the baseline characteristics and clinical outcome between patients with or without prominent FVH in each group, dividing subjects into two groups based on reperfusion therapy. Thereafter, the differences in characteristics and their relation to 90-day outcomes (mRS ≤ 1 vs. mRS ≥ 2) were also analyzed. Multivariate binary logistic regression analysis with a stepwise forward selection of variables was performed to determine independent predictors of an unfavorable outcome at 3 months in patients with mild stroke and large artery occlusion. All variables with a *P* < 0.2 in the univariate analysis were entered in this logistic regression model. Interobserver agreement of FVH grading was analyzed by the κ statistic. A *P* <0.05 was considered statistically significant in all statistical analyses performed using SPSS for Windows, version 23.0 (IBM Corp., Armonk, NY).

## Results

A total of 157 patients who had anterior circulation stroke with mild symptoms and MCA occlusion were admitted to our stroke center during the study period. We excluded 45 of these patients based on the following criteria: (1) admission after 24 h from the last normal time (*n* = 34), (2) unavailable or poor-quality MR images (*n* = 7), and (3) tandem occlusion (*n* = 4). Ultimately, 112 patients [69 men and 43 women; median age, 67 years (IQR, 54–79 years)] were analyzed. The presence of prominent FVH was observed in 80 patients (71.4%, κ = 0.708). The median onset to image time was 433 (192–629) min. Supplementary Table 1 presents the general characteristics and radiologic features of patients who had and had not received reperfusion therapy. Thirty-eight patients were treated with reperfusion therapy and 43 patients (38.4%) had unfavorable outcomes (mRS score 2–6) at 90 days. The median onset to image time was delayed in patients who did not receive thrombolytic therapy (346 min vs. 148 min, *P* < 0.001). This implies that the exclusion of patients from rtPA was indeed based on the breach of the therapeutic time window. Patients with reperfusion therapy had higher NIHSS scores on admission (*P* = 0.044) and earlier onset to image time (*P* < 0.001) than those without reperfusion therapy.

Prominent FVH was significantly more frequent in patients with FVH-DWI mismatch than in those without (Supplementary Table 2). In 74 patients who had not received reperfusion therapy, the proportion of unfavorable outcome at 3 months was significantly higher in the prominent FVH group than in the non-prominent FVH group (44.4 vs. 15.0%, *P* = 0.029). However, the difference of END between patients with prominent FVH and those without was not statistically significant (20 vs. 5%, *P* = 0.162). Among 38 patients who received reperfusion therapy, there were no significant differences in prognosis between the prominent and non-prominent FVH groups (Table 1).

**Table 1:** Clinical and demographic characteristics according to the presence of FVH in patients who had or had not received reperfusion therapy.

	**No reperfusion therapy (*****N*** **= 74)**			**Reperfusion therapy (*****N*** **= 38)**		
	**FVH (-), *N* = 20**	**FVH (+), *N* = 54**	***P*-value**	**FVH (-), *N* = 12**	**FVH (+), *N* = 26**	***P*-value**
Age, median (IQR)	62 (54–72.5)	66.5 (54–78)	0.526	59.5 (42–68)	64.5 (56–73)	0.191
Female, *n* (%)	9 (45)	20 (37)	0.533	5 (41.7)	9 (34.6)	0.675
Risk factors, *n* (%)						
Hypertension	12 (60)	32 (59.3)	0.954	7 (58.3)	14 (53.8)	0.796
Diabetes mellitus	4 (20)	10 (18.5)	0.999	2 (16.7)	6 (23.1)	0.999
Smoking	4 (20)	22 (40.7)	0.110	8 (66.7)	8 (30.8)	0.075
Hyperlipidemia	4 (20)	11 (20.4)	0.999	2 (16.7)	5 (19.2)	0.999
Atrial fibrillation	3 (15)	19 (35.2)	0.151	2 (18.2)	12 (48.0)	0.142
Pervious stroke	2 (10)	10 (18.5)	0.494	3 (25)	5 (19.2)	0.689
SBP on arrival, mm Hg, median (IQR)	130 (120–155)	130 (110–150)	0.431	145 (135–165)	140 (130–150)	0.715
DBP on arrival, mm Hg, median (IQR)	80 (80–90)	80 (70–90)	0.274	95 (80–100)	90 (80–90)	0.569
Glucose level, mg/dL,	111 (99–132)	116 (101–146)	0.495	116 (108–152)	122 (111–135)	0.849
NIHSS score on admission, median (IQR)	2.5 (1.5–4)	2.5 (2–4)	0.456	3.5 (2–4.5)	4 (2–5)	0.919
DWI lesion, mL, median (IQR)	3.36 (0.18–10.3)	7.1 (1.54–25.7)	0.193	4.34 (1.8–12.5)	6.97 (1.27–15.5)	0.457
Time from symptom onset to image, min, median (IQR)	433 (192–629)	346 (152–720)	0.580	80 (57–127)	61 (50–100)	0.395
Onset to rt–PA time, min, median (IQR)				122 (85–178)	108 (83–160)	0.719
Reperfusion therapy, *n* (%)						0.692
Intravenous rtPA alone				10 (83.3)	19 (73.1)	
Intravenous rtPA + endovascular				1 (8.3)	5 (19.2)	
Primary endovascular				1 (8.3)	2 (7.7)	
FVH–DWI mismatch, *n* (%)	5 (25)	36 (66.7)	0.001	5 (41.7)	21 (80.8)	0.016
Occlusion location, *n* (%)			0.872			0.307
Right	13 (65)	34 (63)		4 (33.3)	14 (53.8)	
Left	7 (35)	20 (37)		8 (66.7)	12 (46.2)	
Occlusion site, *n* (%)			0.387			0.525
M1 proximal	10 (50)	19 (35.2)		7 (58.3)	12 (46.2)	
M1 distal	6 (30)	16 (29.6)		3 (25)	5 (19.2)	
M2	4 (20)	19 (35.2)		2 (16.7)	9 (34.6)	
END, *n* (%)	1 (5)	11 (20.4)	0.162	3 (25)	3 (11.5)	0.357
Unfavorable outcome, *n* (%)	3 (15.0)	24 (44.4)	0.029	6 (50)	10 (38.5)	0.503

*FVH, fluid–attenuated inversion recovery vascular hyperintensity; IQR, interquartile range; SBP, systolic blood pressure, DBP, diastolic blood pressure; NIHSS, National Institutes of Health Stroke Scale; DWI, diffusion-weighted image; rtPA, recombinant tissue plasminogen activator; END, early neurological deterioration*.

Regarding the prognostic analysis of patients who had not received reperfusion therapy, higher NIHSS scores (*P* = 0.006), initial larger DWI volume (*P* = 0.023), and prominent FVH (*P* = 0.029) were more frequently observed in patients with an unfavorable outcome in the univariate analysis. Diabetes mellitus and location of the occlude vessel differed between the two groups at *P* < 0.20 and were included in multivariable logistic regression analysis. Multivariate logistic regression analysis showed that higher NIHSS scores on admission [odds ratio [OR] = 1.67; 95% confidence interval [CI], 1.16–2.41; *P* = 0.006], proximal MCA occlusion [OR = 7.31; 95% CI, 1.68–31.9; *P* = 0.008], and prominent FVH [odds ratio = 5.49; 95% CI, 1.29–23.4; *P* = 0.021] were independently associated with an unfavorable outcome (Table 2).

**Table 2:** Characteristics of the study population according to outcome at 3 months and multivariable analysis for an unfavorable outcome at 90 days.

	**Favorable outcome (mRS ≤ 1, *N* = 47)**	**Unfavorable outcome (mRS > 1, *N* = 27)**	***P*-value**	**Adjusted OR**	***P*-value**
Age, median (IQR)	64 (53–77)	67 (54–79)	0.354		
Female, *n* (%)	18 (38.3)	11 (40.7)	0.836		
Hypertension, *n* (%)	27 (57.4)	17 (63)	0.642		
Diabetes mellitus, *n* (%)	6 (12.8)	8 (29.6)	0.075		
Atrial fibrillation, *n* (%)	16 (34)	6 (22.2)	0.284		
Smoking, *n* (%)	18 (38.3)	8 (29.6)	0.452		
Hyperlipidemia, *n* (%)	12 (25.5)	3 (11.1)	0.229		
Previous stroke, *n* (%)	6 (12.8)	6 (22.2)	0.288		
SBP on arrival, mm Hg, median (IQR)	130 (120–150)	130 (120–180)	0.421		
DBP on arrival, mm Hg, median (IQR)	80 (70–90)	80 (80–100)	0.289		
Baseline NIHSS score, median (IQR)	2 (1–4)	4 (2–5)	0.006	1.67 (1.16–2.41)	0.006
DWI volume, mL, median (IQR)	2.94 (0.43–16.3)	8.81 (4.14–31.38)	0.023		
Site, *n* (%)			0.669		
Right	29 (61.7)	18 (66.7)			
Left	18 (38.3)	9 (33.3)			
Location, *n* (%)			0.142		0.030
M2	18 (38.3)	5 (18.5)		Ref	
Distal M1	14 (29.8)	8 (29.6)		2.87 (0.68–12.2)	0.152
Proximal M1	15 (31.9)	14 (51.9)		7.31 (1.68–31.9)	0.008
FVH, *n* (%)	30 (63.8)	24 (88.9)	0.029	5.49 (1.29–23.4)	0.021

*SBP, systolic blood pressure; DBP, diastolic blood pressure; DWI, diffusion-weighted image, FVH, fluid-attenuated inversion recovery vascular hyperintenisty*.

## Discussion

Our study showed that a higher NIHSS, proximal vessel occlusion, and the presence of prominent FVH were all independent predictors of unfavorable outcome at 3 months in patients with mild stroke who had not received reperfusion therapy. The prognostic value of FVH differed according to the use of reperfusion therapy. Prominent FVH was associated with an unfavorable outcome in patients who had not received reperfusion therapy, but not in patients who received reperfusion therapy.

FVH was noted in 45% of cases in the MR images obtained within 24 h of neurological symptom onset (18). However, the prognostic value of FVH for stroke outcomes is still controversial (9–17). The contrasting results of previous studies may stem from heterogeneous study populations with respect to large artery occlusion and reperfusion therapy. Some studies included all ischemic stroke patients without regard to large artery occlusion and investigated only the presence of FVH without measurement of FVH burden. Under these conditions, FVH can predict proximal arterial occlusions and initially more severe strokes (9–11). Conversely, other authors reported that FVH, which was surrogate marker of ischemic penumbra, was associated with favorable outcomes after reperfusion therapy in patients with large artery occlusion (12–15). However, among patients with acute ischemic stroke with large vessel steno-occlusion who had not received reperfusion therapy, those with FVH were more likely to have END and poor outcome (16, 17).

We evaluated the association between prominent FVH and clinical outcome, considering whether reperfusion therapy was administered, in selective mild stroke with MCA occlusion. For patients with mild symptoms and MCA occlusion who did not receive reperfusion therapy, those with prominent FVH were more likely to have an unfavorable outcome compared to those without prominent FVH. These finding is consistent with previous studies (16, 17). FVH indicates retrograde leptomeningeal collaterals (6) and is a surrogate marker of perfusion-diffusion mismatch in hyperacute stroke (7, 12, 19). The extent of FVH is correlated with perfusion-diffusion mismatch volume in large artery occlusion (7, 13, 16) as well as with stroke severity, initial lesion volume, and the severity of hypoperfusion (20). In our study, there was about 50% increase in DWI lesion size in patients with prominent FVH, although this was not statistically significant (Supplementary Table 2). If blood flow is sufficient to overcome hypoperfusion, peripheral collateral circulations can prolong tissue survival. However, as FVH implies sluggish flow, a region of insufficient collateralization could develop infarct growth over time (17, 20). In spite of the presence of large artery occlusion, our subjects seemed to have a small DWI volume due to the restriction of enrollment to minor stroke patients with NIHSS ≤5. Thus, prominent FVH possibly represented a small DWI volume and large mismatch region in our study. This explains why patients with prominent FVH are more likely to have FVH-DWI mismatch and an unfavorable outcome when not treated with reperfusion therapy.

In contrast, some studies reported that the presence of prominent FVH or FVH-DWI mismatch is an early prognostic marker of good outcome after thrombolytic therapy or successful recanalization by endovascular treatment in hyperacute stroke patients with proximal MCA occlusion (12–15). There is a possibility that reperfusion therapy diminished the mismatched area with prominent FVH unlike in the non-reperfusion therapy group. Thus, FVH, which represents penumbra by leptomeningeal collaterals, could be a hallmark of a negative outcome in the absence of reperfusion therapy or a positive outcome in case of reperfusion therapy (21). However, in our study, we did not find the association between prominent FVH and stroke outcome in patients who received reperfusion therapy to be statistically significant. There was no association between reperfusion therapy and function outcome in patients with prominent FVH (Supplementary Table 3). This might have been due to the extremely low number of patients in the reperfusion therapy group.

Recent randomized clinical trial failed to demonstrate the efficacy of intravenous alteplase in acute ischemic stroke patients with NIHSS scores of 0 to 5 (22). Current guidelines recommend endovascular treatment only for patients with large artery occlusion in anterior circulation who have an NIHSS score of ≥6 (23). However, the benefit of endovascular treatment for acute mild ischemic stroke (NIHSS < 6) with large vessel occlusion is uncertain (24, 25). In the future, studies for an adequate reperfusion strategy for mild stroke patients with prominent FVH and MCA occlusion may be necessary.

The present study has several important limitations. First, this study was retrospective with a small sample size, using clinical registry data of a single hospital. Thus, bias in patient selection could have occurred. Second, in this study, patients without prominent FVH who received reperfusion therapy had unexpectedly more frequent unfavorable outcomes among the four groups. However, this observation might not be significant because the number of patients in this subgroup was very small and 3 out of 6 patients with unfavorable outcome suffered one symptomatic intracranial hemorrhage after thrombolysis and two recurrent ischemic stroke within 3 months after onset. The number of patients with reperfusion therapy was also small. An association between FVH and outcome could have been missed because of a lack of power. Third, follow-up MRI scans were not conducted in most of the patients treated with reperfusion therapy because of the CT follow-up protocol during the study period. Therefore, the presence of recanalization and change of infarct size after reperfusion therapy were not evaluated. Lastly, we could not measure pretreatment perfusion-diffusion mismatch volume. However, FVH-DWI mismatch may identify patients with proximal occlusion most likely to benefit from recanalization (12). Thus, FVH-DWI mismatch may be a simple approach for predicting the presence of penumbra in our study.

In summary, in a group of patients who do not receive reperfusion therapy, those with prominent FVH are more likely to have an unfavorable outcome than those without prominent FVH. Our findings may help select the patients who should receive reperfusion therapy or hemodynamic control in this group.

## Data Availability

The datasets generated during the current study are available from the corresponding author on reasonable request.

## Author Contributions

D-HK: conception and design, and drafting the manuscript, and revising it critically for important intellectual content. Y-KL: conception and design, acquisition of data, and drafting the manuscript. J-KC: data collection and data interpretation. All authors have read and approved the final manuscript.

### Conflict of Interest Statement

The authors declare that the research was conducted in the absence of any commercial or financial relationships that could be construed as a potential conflict of interest.
