# Supplementary material for: Prominent FLAIR Vascular Hyperintensity Is a Predictor of Unfavorable Outcomes in Non-thrombolysed Ischemic Stroke Patients With Mild Symptoms and Large Artery Occlusion
Source: Front Neurol. 2019 Jul 2;10:722. doi: 10.3389/fneur.2019.00722 (PMC6614286; doi:10.3389/fneur.2019.00722)
Supplement: Supplementary file 1 [file Table_1.DOCX]

Supplemental material

Supplementary Table 1. General characteristics of the subjects according to reperfusion therapy.

|  | All (N=112) | No reperfusion therapy (N=74) | Reperfusion therapy (N=38) | P-value |
| --- | --- | --- | --- | --- |
| Age, median (IQR) | 67(54-79) | 65(54-77) | 64.5(54-71) | 0.363 |
| Female, n (%) | 43(38.4) | 29(39.2) | 14(36.8) | 0.809 |
| Risk factors, n (%) |  |  |  |  |
| Hypertension | 65(58) | 44(59.5) | 21(53.3) | 0.670 |
| Diabetes mellitus | 22(19.6) | 14(18.9) | 8(21.1) | 0.788 |
| Smoking | 42(37.5) | 26(35.1) | 16(42.1) | 0.471 |
| Hyperlipidemia | 22(19.6) | 15(20.3) | 7(18.4) | 0.816 |
| Atrial fibrillation | 36(32.1) | 22(29.7) | 14(36.8) | 0.445 |
| Pervious stroke | 20(17.9) | 12(16.2) | 8(21.1) | 0.527 |
| SBP on arrival, mm Hg, median (IQR) | 140(120-180) | 130(120-150) | 140(130-160) | 0.110 |
| DBP on arrival, mm Hg, median (IQR) | 90(80-100) | 80(70-90) | 90(80-100) | 0.080 |
| Glucose level, mg/dL, | 122(105-208) | 114.5(101-133) | 122(108-141) | 0.309 |
| NIHSS score on admission, median ( IQR) | 4(2-5) | 2.5(2-4) | 4(2-5) | 0.044 |
| DWI lesion, mL, median (IQR) | 8.69(2.19-35,4) | 6.55(1.54-20.51) | 5.38(1.27-16,16) | 0.882 |
| Time from symptom onset to image, min, median (IQR) | 236(130-507) | 378(168-720) | 147(95-231) | <0.001 |
| Prominent FVH, n(%) | 80(71.4) | 54(73) | 26(68.4) | 0.614 |
| FVH-DWI mismatch, n(%) | 67(59.8) | 41(55.4) | 26(68.4) | 0.183 |
| Occlusion location, n(%) |  |  |  | 0.101 |
| Right | 65(58) | 47(63.5) | 18(47.4) |  |
| Left | 47(42) | 27(36.5) | 20(52.6) |  |
| Occlusion site, n(%) |  |  |  | 0.490 |
| M1 proximal | 48(42.9) | 29(39.2) | 19(50) |  |
| M1 distal | 30(26.8) | 22(29.7) | 8(21.1) |  |
| M2 | 34(30.4) | 23(31.1) | 11(28.9) |  |
| Early neurological deterioration, n (%) | 18(16.1) | 12(16.2) | 6(15.8) | 0.954 |
| Unfavorable outcome, n (%) | 42(38.4) | 27(36.5) | 16(42.1) | 0.563 |

IQR, interquartile range; SBP, systolic blood pressure; DBP, diastolic blood pressure; NIHSS, National Institutes of Health Stroke Scale; DWI, diffusion-weighted image; FVH, fluid-attenuated inversion recovery vascular hyperintensity

Supplementary Table 2. General characteristics of the subjects according to their FVH status.

|  | No prominent FVH (N=32) | Prominent FVH  (N=80) | P-value |
| --- | --- | --- | --- |
| Age, median (IQR) | 62(50-72) | 65(54-76) | 0.220 |
| Female, n (%) | 18(56.3) | 51(63.7) | 0.461 |
| Risk factors, n (%) |  |  |  |
| Hypertension | 19(59.4) | 46(57.5) | 0.856 |
| Diabetes mellitus | 6(18.8) | 16(20) | 0.880 |
| Smoking | 12(37.5) | 30(37.5) | <0.999 |
| Hyperlipidemia | 6(18.8) | 16(20) | 0.880 |
| Atrial fibrillation | 5(15.6) | 31(38.8) | 0.018 |
| Pervious stroke | 5(15.6) | 15(18.8) | 0.696 |
| SBP on arrival, mm Hg, median (IQR) | 140(125-160) | 130(120-150) | 0.255 |
| DBP on arrival, mm Hg, median (IQR) | 85(80-100) | 80(70-90) | 0.176 |
| Glucose level, mg/dL, | 111(102-137) | 117(104-141) | 0.666 |
| NIHSS score on admission, median (IQR) | 3(2-4) | 3(2-5) | 0.468 |
| DWI lesion, mL, median (IQR) | 4.33(1.05-7.6) | 7.1(1.63-23.3) | 0.101 |
| Time from symptom onset to MRI, min, median (IQR) | 238(147-510) | 233(125-402) | 0.954 |
| FVH-DWI mismatch, n(%) | 10(31.3) | 57(71.3) | <0.001 |
| Occlusion location, n(%) |  |  | 0.505 |
| Right | 17(53.1) | 48(60) |  |
| Left | 15(46.9) | 32(40) |  |
| Occlusion site, n(%) |  |  | 0.210 |
| M1 proximal | 17(53.1) | 31(38.8) |  |
| M1 distal | 9(28.1) | 21(26.3) |  |
| M2 | 6(18.8) | 28(35) |  |

IQR, interquartile range; SBP, systolic blood pressure; DBP, diastolic blood pressure; NIHSS, National Institutes of Health Stroke Scale; DWI, diffusion-weighted image; FVH, fluid-attenuated inversion recovery vascular hyperintensity

Supplementary Table 3. General characteristics according to reperfusion therapy among the patients with prominent FVH

|  | No reperfusion therapy + prominent FVH (N=54) | Reperfusion therapy + prominent FVH  (N=26) | P-value |
| --- | --- | --- | --- |
| Age, median (IQR) | 66.5(54-78) | 64.5(56-73) | 0.644 |
| Female, n (%) | 20(37) | 17(65.4) | 0.833 |
| Risk factors, n (%) |  |  |  |
| Hypertension | 32(59.3) | 14(53.8) | 0.646 |
| Diabetes mellitus | 10(18.5) | 6(23.1) | 0.633 |
| Smoking | 22(40.7) | 8(30.8) | 0.388 |
| Hyperlipidemia | 11(20.4) | 5(19.2) | 0.905 |
| Atrial fibrillation | 19(35.2) | 12(46.2) | 0.346 |
| Pervious stroke | 10(18.5) | 5(19.2) | 0.939 |
| SBP on arrival, mm Hg, median (IQR) | 130(110-150) | 140(130-150) | 0.221 |
| DBP on arrival, mm Hg, median (IQR) | 80(70-90) | 90(80-90) | 0.148 |
| Glucose level, mg/dL, | 116(103-146) | 122(111-135) | 0.583 |
| NIHSS score on admission, median (IQR) | 2.5(2-4) | 4(2-5) | 0.116 |
| DWI lesion, mL, median (IQR) | 7.1(1.91-28.7) | 6.98(1.27-17.9) | 0.937 |
| Time from symptom onset to MRI, min, median (IQR) | 346(152-720) | 148(107-231) | <0.001 |
| FVH-DWI mismatch, n(%) | 36(66.7) | 21(80.8) | 0.192 |
| Occlusion location, n(%) |  |  | 0.436 |
| Right | 34(63) | 14(53.8) |  |
| Left | 20(37) | 12(46.2) |  |
| Occlusion site, n(%) |  |  | 0.530 |
| M1 proximal | 19(35.2) | 12(46.2) |  |
| M1 distal | 16(29.6) | 5(19.2) |  |
| M2 | 19(35.2) | 9(34.6) |  |
| Early neurological deterioration, n (%) | 11(20.4) | 3(11.5) | 0.531 |
| Unfavorable outcome, n (%) | 24(44.4) | 10(38.5) | 0.612 |

IQR, interquartile range; SBP, systolic blood pressure; DBP, diastolic blood pressure; National Institutes of Health Stroke Scale; DWI, diffusion-weighted image; FVH, fluid-attenuated inversion recovery vascular hyperintensity
